# Hotspot exons are common targets of splicing perturbations

**DOI:** 10.1038/s41467-021-22780-2

**Published:** 2021-05-12

**Authors:** David T. Glidden, Jeramiah L. Buerer, Camillo F. Saueressig, William G. Fairbrother

**Affiliations:** 1 Center for Computational Molecular Biology, Brown University, Providence, RI USA; 2 Molecular Biology, Cell Biology and Biochemistry, Brown University, Providence, RI USA; 3 Hassenfeld Child Health Innovation Institute of Brown University, Providence, RI USA

**Keywords:** Gene expression profiling, Gene regulation, RNA splicing

## Abstract

High-throughput splicing assays have demonstrated that many exonic variants can disrupt splicing; however, splice-disrupting variants distribute non-uniformly across genes. We propose the existence of exons that are particularly susceptible to splice-disrupting variants, which we refer to as hotspot exons. Hotspot exons are also more susceptible to splicing perturbation through drug treatment and knock-down of RNA-binding proteins. We develop a classifier for exonic splice-disrupting variants and use it to infer hotspot exons. We estimate that 1400 exons in the human genome are hotspots. Using panels of splicing reporters, we demonstrate how the ability of an exon to tolerate a mutation is inversely proportional to the strength of its neighboring splice sites.

## Abstract (web summary)

Splicing-disrupting mutations are linked to diseases. By employing a machine learning approach, the authors show that certain exons, termed hotspot exons, are enriched for splicing-disruption variants and susceptible to splicing perturbations.

## Introduction

Recent studies have shown that splice-disrupting variants are strongly linked to disease, with up to one-third of disease-causing variants altering splicing^1,2^. This discovery underscores the importance of determining splice-disrupting potential when classifying novel variants in terms of their pathogenicity. Recent technological advances, such as high-throughput splicing assays and machine learning tools, can classify potential splice-disrupting variants at the same throughput and scale of variant discovery^3–5^. Despite these advances, little progress has been made in exploiting these findings to develop drugs that rescue splicing in specific variants. The first drug developed to target a splicing disorder is nusinersen. It is an antisense oligonucleotide (ASO) that silences a splicing element in *smn2* in order to treat spinal muscular atrophy^6–8^. However, allele-specific therapies face an economic conundrum. Because there are thousands of potential splice disrupting variants, it is unlikely that additional ASOs will be developed to target each one, given the time and cost of drug development.

Most drug development costs can be averted by repurposing existing drugs. RNA-seq could facilitate drug repurposing for splicing disorders by detecting all of the splicing events caused by drug treatment. In fact, many Food and Drug Administration (FDA)-approved drugs have already been shown to affect splicing as an off-target effect. It is possible that some of these effects may, by chance, rescue or exacerbate a splicing disorder. To date, however, none of these drugs have been repurposed for splicing disorders^9–12^. With a better understanding of splicing regulation, RNA-seq could help to discover potential splicing targets for other FDA-approved drugs. In addition, when drugs are found to exacerbate a splicing disorder, warnings could be issued for patients carrying a corresponding splice-disrupting variant.

Here, we used a machine learning approach to prioritize the key determinants of exon recognition. We find that exonic features are better predictors of splice-disrupting variants than more local features such as which ESE motif is disrupted. A certain subset of exons (i.e.,“hotspot exons”) are prone to exon skipping. Subsequent analyses show that hotspot exons are (1) enriched for splice-disrupting variants, (2) dependent on a high number of RNA-binding protein (RBP) binding events, and (3) sensitive to drug treatment. Mathematical models of splicing kinetics, as well as mutagenesis studies in FAS exon 6, have suggested that exon skipping is sensitive to epistatic effects between subsequent exonic mutations and demonstrates a wild-type exon’s inclusion level is a predictor of a mutation’s ability to disrupt splicing^13^. Similar studies in RON exon 11 have shown exon skipping is also sensitive to cooperative RBP binding^14^. We report evidence of both phenomena in vivo across the human exome. Lastly, we show that exon skipping can be rescued by repressing the recognition of a downstream flanking exon. Using hotspot exons, we can predict where splice-disrupting variants are likely to be discovered, and outline a strategy for developing a class of drugs that targets flanking exons to partially rescue the effect of splice-disrupting variants.

## Results

### Exonic features predict splice-disrupting variants

**Fig. 1:**
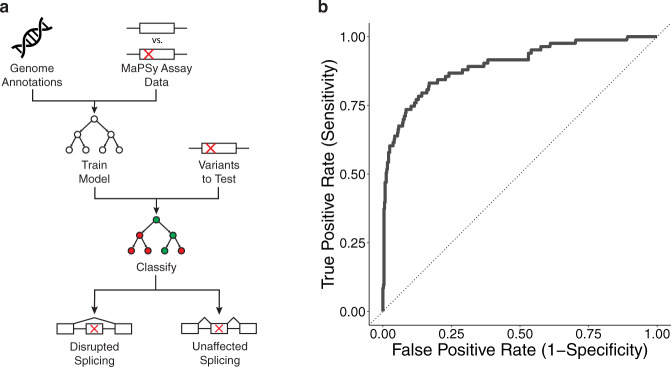
Prediction workflow. **a** Computational pipeline of the GBM classifier. The classifier is composed of features extracted from genomic sequence and features related to variants tested by MaPSy. **b** Receiver–operator characteristic (ROC) curve for the GBM classifier.

Recently, a high-throughput assay (MaPSy) was developed to measure the degree of splicing disruption caused by SNVs, reported as the allelic ratio. A variant is determined to be splice-disrupting when ${{|}{\log }}_{2}\left({\rm{allelic}}\; {\rm{ratio}}\right)|\ge 1.5$ (FDR < 0.05). Using data from MaPSy as well as whole-genome annotations, a gradient boosting machines (GBM) classifier was trained to identify splice-disrupting variants (AUC = 0.93) (Fig. 1)^15–17^.

**Fig. 2:**
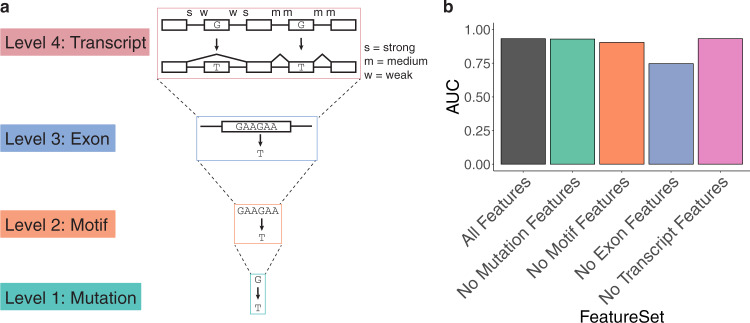
Hierarchy of features for variant prediction. **a** Features are categorized into one of four groups: mutation, motif, exon, and transcript. Examples of mutation features include the specific base pair change (G → T mutations disrupt splicing above all others), and whether or not the wild-type or mutant allele is predicted to be hybridized. Examples of motif features include change in Maxent score, and the changes in cis-element scores for hexamers affected by the mutation. Examples of exonic features include splice site usage and average EI score for all hexamers in the exon. Examples of transcript features include the number of exons in the transcript, and gene length. **b** Bar plot of the area under the curve (AUC) for different iterations of the GBM classifier with certain feature groups excluded.

In addition to predicting individual events, the classifier was also used to determine which features contribute the most to a successful prediction. To gain this sort of mechanistic information from the classifier, we characterized the relative contributions of the features of the GBM classifier through a process called feature selection. When several features are highly correlated, it is more challenging to evaluate the relative contribution of individual features^18^. To circumvent this problem, broad categories of features were defined in increments of increased scope (i.e., mutation <  motif < exon < transcript) (Fig. 2a). These categories were then ranked by their contribution to the accuracy of the classifier. In order to perform this ranking, the GBM classifier was retrained multiple times, excluding a different feature category each time. Only the removal of exonic features resulted in a loss of performance (Fig. 2b). Similar results were observed when using a Random Forest classifier (Fig. S1)^19^. In addition, when training the GBM with only one feature group at a time, the exonic features showed the highest AUC (Fig. S2). These results suggest that variants themselves and the binding motifs they disrupt hold little predictive information. Instead, it is the properties of the exons that determine which variants disrupt splicing.

### Variants in the same exon tend to affect splicing similarly

**Fig. 3:**
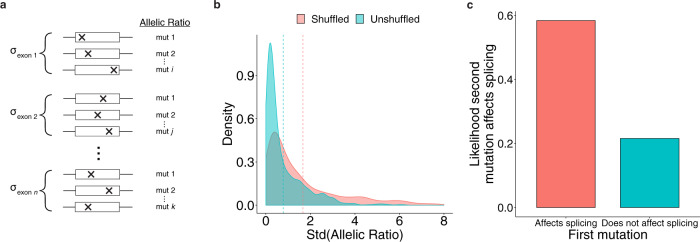
Variants within the same exon are likely to have similar allelic ratios. **a** Exons from the MaPSy study were selected if they contained more than one variant which was assayed for allelic ratio (*n* = 1017). The standard deviation of allelic ratio was calculated for each exon. **b** Distributions of the standard deviations before and after shuffling allelic ratio values across all exons. Dashed lines represent the median values for the unshuffled (left) and shuffled (right) distributions. **c** Conditional probabilities that a randomly sampled exonic mutation will affect splicing depending on whether or not a previously sampled mutation in the same exon affects splicing.

If an exon contains most of the information that determines susceptibility to exon skipping, then most mutations in an exon should affect splicing in a similar way. In other words, the measurements of allelic ratios (i.e., the difference in mut/wildtype ratio in the DNA vs. the RNA) should be similar for variants drawn from the same exon. In order to test this idea, we examined exons that were previously assayed in the MaPSy study. Exons were selected only if more than one variant was assayed in them (i.e., there are multiple mutant alleles, which have corresponding allelic ratios from the assay) (*n* = 1017). This criterion allowed for the computation of the standard deviation of the allelic ratios for each exon (Fig. 3a). The distribution of these allelic ratios was then tested against the null hypothesis, which is that variants in the same exon do not have similar allelic ratios. The null hypothesis model was created by randomly shuffling allelic ratios across all exons, thereby removing any potential dependencies between variants tested in the same exon. The standard deviations in the unshuffled distribution containing the true allelic ratios were significantly lower than those in the shuffled one (*p* « 0.0001; Wilcoxon signed rank test) (Fig. 3b). This observation is consistent with the findings from the GBM classifier (Fig. 2b), which suggest that exonic features are strong predictors of allelic ratio. In addition to exonic features, it also indicates that the allelic ratio from one variant may be a valuable predictor of allelic ratios from other variants in the same exon. To determine its predictive power, two assayed mutations were selected from each exon. When the first mutation affects splicing, the probability that the second mutation will also affect splicing increases by 0.37 (0.58 total) (Fig. 3c).

### RBP-binding sites required for splicing cluster in hotspot exons

As hotspot exons are more sensitive to variants that disrupt splicing, it was hypothesized that they would also be more susceptible to changes in RBP-binding events. RBP-binding events are not guaranteed to have an impact on splicing. In order to determine the RBP-binding events that each exon requires for splicing, RNA-seq data from both siRNA knockdown and eCLIP studies from ENCODE was analyzed^20,21^. The HepG2 and K562 cell lines were selected, because they had a total of 35 and 31 splicing factors studied in ENCODE, respectively, using both methods. In both cell lines, ~10% of the eCLIP binding sites were deemed necessary for splicing of an exon. We term these loci “functional binding sites” and define them as sites in or near an exon whose splicing is affected by corresponding RBP knockdown.

**Fig. 4:**
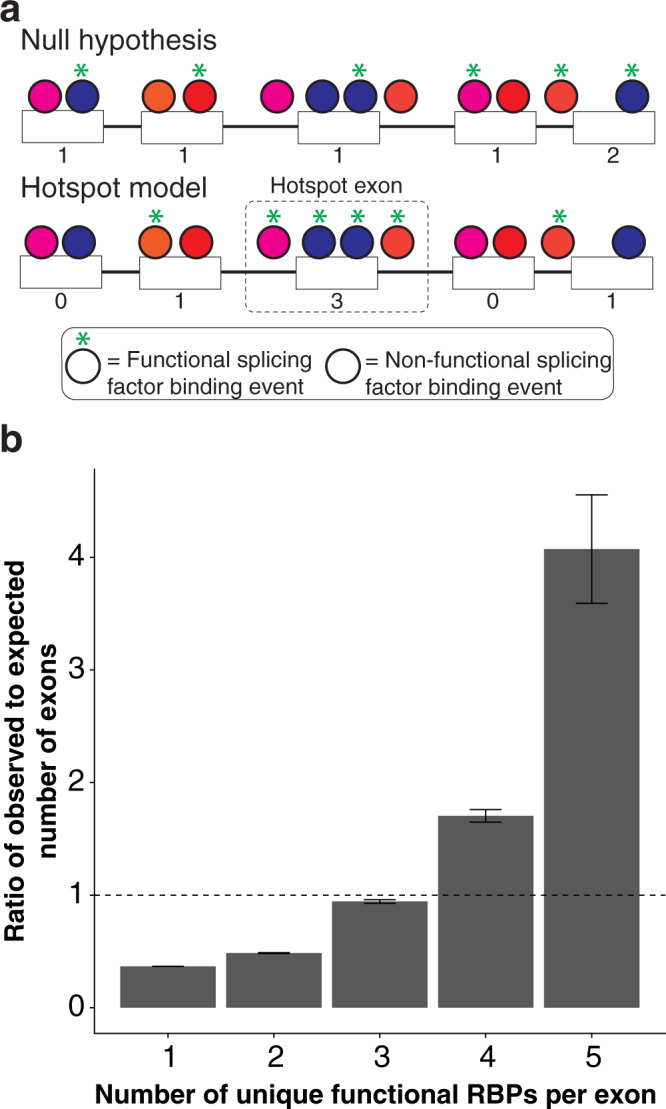
Hotspot exons depend on several RBP-binding events. **a** Hotspot exons tend to rely on multiple RBPs for correct splicing. Numbers below exons indicate how many unique RBPs are required for the splicing of the exon. Circles of the same color represent the same RBP. **b** Enrichment above expectation in the number of exons with various numbers of unique RBPs necessary for splicing (*n* = 3505 binding sites). Data are presented as mean ± SEM.

To determine whether functional binding sites cluster in hotspot exons like splice-disrupting variants do, the distribution of observed functional binding sites per exon was compared to a theoretical distribution of the total number of expected binding sites for each exon. The theoretical distribution was created by sampling from the set of “non-functional binding sites” (no effect on splicing) at the level of the set of functional binding sites (*n* = 3505) (Fig. 4a). This sampling approach was used to model the tendency for RBP-binding sites to cluster, because it should capture all the sequence and expression biases that could confound other approaches. Compared to the theoretical distribution, the observed distribution of functional binding sites is enriched for exons with higher numbers of bound RBPs (Fig. 4b). That is, there are fewer exons than expected with 1 or 2 functional binding sites, but more than expected with high numbers of functional binding sites. This analysis suggests that functional binding sites cluster in hotspot exons, just as splice-disrupting variants do. This phenomenon is further demonstrated by performing an analysis similar to the prior analysis of splicing mutations (Fig. 3c). By randomly sampling two eCLIP sites from exons with more than one binding event, the probability that the second sampled site in the same exon affects splicing increases by 0.15–0.20 when the first site has an effect on splicing (Fig. S3). Thus, the presence of a functional binding site provides information on the effect of another site in the same exon, much like how a splice-disrupting variant can predict the splicing effect of another sampled variant.

### Hotspot exons have low relative splice site usage

If additional hotspot exons can be identified in the genome, novel variants discovered in these exons could be prioritized for further studies, such as MaPSy or other functional assays. Although these assays are designed to be high-throughput, they cannot yet assay all exons simultaneously. This limitation can be addressed by a priori elimination of exons whose splicing is predicted to be unaffected by perturbations. Prior work has shown that exons with weak splice sites are susceptible to splicing perturbations^3,22^. However, these results came from massively parallel splicing assays, which were limited to the analysis of a few thousand exons. In order to generalize the prediction of potential splicing perturbations across the entire genome without relying on reporter assays, an empirical measure of splice site recognition was sought. Splice site usage—the frequency at which exons splice at particular loci—can be measured from WT RNA-seq data. Of the 287,410 splice sites for which usage was calculated in the K562 cell line, 71% (202,975) had a usage of 1.0. The usages of the remaining splice sites are distributed bimodally around 0 and 1 with 51.2% (43,249) of non-constitutive splice sites having usages greater than 0.9 and 20.6% (17,373) having usages less than 0.1 (Fig. S4).

**Fig. 5:**
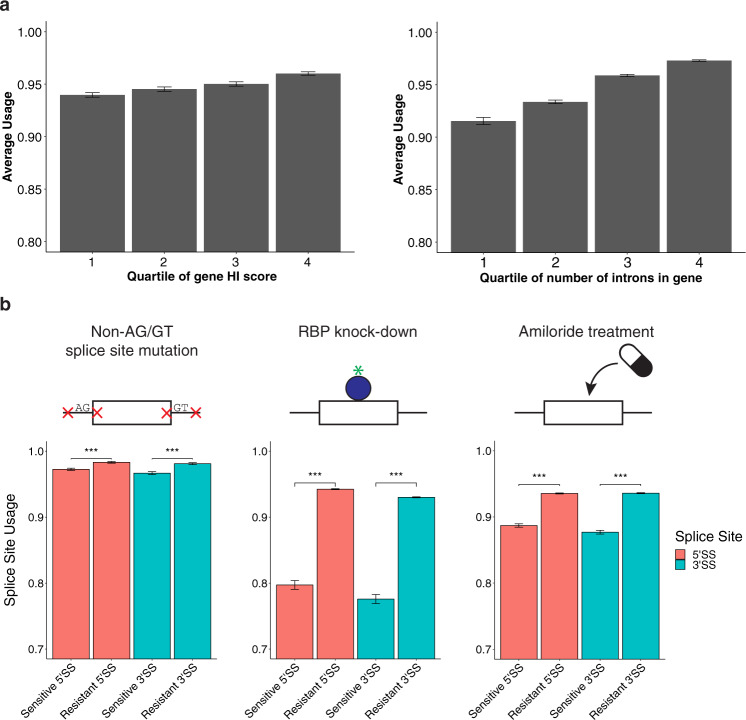
Hotspot exons have lower splice site usage. **a** Exons in the ENCODE HepG2 dataset with splice site usage values were binned into quartiles based on either the haploinsufficiency score (left, *n* = 6660 exons) or the number of introns (right, *n* = 9558 exons) in their respective genes. Data are presented as mean ± SEM. **b** Three separate analyses were performed on splice site usage. In the first case (left), exons were categorized based on the presence of a splice site mutation reported in HGMD that lies outside of the essential AG/GT motif in canonical 5′ and 3′ splice sites (*n* = 3600 exons, *p* = 1.912e−8 for 5′SS and *p* = 1.414e−9 for 3’ss (one-sided Mann–Whitney test)). In the second case (middle), exons were categorized based on whether or not splicing was affected in an ENCODE RBP knockdown study (*n* = 2333 exons, *p* < 2.2e−16 for both 5’SS and 3’SS (one-sided Mann–Whitney test)). In the last case (right), exons were categorized based on the effect of amiloride treatment on exon skipping (*n* = 6820 exons, *p* < 2.2e−16 for both 5’SS and 3’SS (one-sided Mann–Whitney test)). The average splice site usage in each case is plotted. Data are presented as mean ± SEM, and *p* values are adjusted for multiple comparisons.

In order to test the validity of splice site usage as a metric, the relationships between splice site usage and gene features relevant to splice sites were examined. The haploinsufficiency (HI) score is one such feature that reflects the proportion of correctly-processed transcripts required for gene function. If a gene is haploinsufficient, it means that having only one copy of the healthy allele is not enough to produce a healthy phenotype^23^. It was hypothesized that the proportion of correctly spliced transcripts is correlated with the joint probability of all necessary splicing events occurring together. Therefore, splice site usage should be positively associated with HI score as well, since haploinsufficient genes are under greater selective pressure to splice correctly. This hypothesis was confirmed by examining the average splice site usage in genes with different levels of HI scores (Fig. 5a, left panel). Likewise, splice site usage should also correlate with the number of introns in a gene. In order to maintain the same joint probability of splicing, the individual splice sites in a gene with numerous introns should be stronger than those in another gene with fewer introns. This tendency was also observed, as genes with more introns have higher average splice site usage than genes with fewer introns (Fig. 5a, right panel). These results suggest splice site usage performs well as an empirical measure of splice site strength; however, it has not been well-established that splice site usage predicts whether different types of perturbations disrupt splicing.

To test the hypothesis that splice site usage is predictive of susceptibility to splicing perturbations, three distinct classes of perturbations were defined: (a) *cis*-mutations, (b) RBP knockdowns, and (c) drug treatment. For each analysis, exons were sorted into sensitive and resistant categories, depending on whether their splicing was altered by a particular perturbation. In the case of *cis*-mutations, the sensitive category was defined as exons with reported splice-disrupting mutations within splice sites, outside of the critical AG/GT dinucleotides. The resistant category contained exons whose splice site mutations were only located in the AG/GT dinucleotides. Comparing these two categories showed that exons which are sensitive to splicing mutations had a lower average splice site usage than exons that are resistant to such mutations (Fig. 5b, left panel). In the case of RBP knockdowns, the sensitive category was composed of exons with functional RBP binding sites (as defined above), and the resistant category contained exons with non-functional binding sites. These two categories also exhibited differences in splice site usage, with exons that were sensitive to RBP knockdown having a lower average usage (Fig. 5b, middle panel). In comparison to mutation-sensitive exons, exons that were sensitive to RBP knockdown had significantly lower usage (*p* value « 0.0001). One possible explanation is the targets of RBPs are frequently alternatively spliced exons; however, switch score analysis does not support this conclusion (Fig. S5). Lastly, the relationship between splice site usage and an exon’s susceptibility to drug treatment was examined. Amiloride was chosen as an exemplar because previous studies have shown that it affects splicing in a large number of genes, some of which are involved in disease pathways such as cancer^10,24^ Again, splice site usage was found to be lower in exons that were sensitive to disruption by amiloride treatment than those whose splicing was resistant to the treatment (Fig. 5b, right panel). These analyses indicate that lower splice site usage appears to be a strong predictor of hotspot exons, which have a generalized susceptibility to splicing perturbations.

Because of the correlation between splice site usage and characteristics of a hotspot exon, and the fact that it is measured from wild-type RNA-seq data, splice site usage can help determine hotspot exons even if no variants of those exons have been assayed for splicing disruption. We created a UCSC Genome Browser track that reports splice site usage. Because splice site usage can vary greatly among tissues, we report the average usage across 19 ENCODE cell lines and limit the reported splice sites to those with low variance (<0.001). See the “Data Availability” section for further information.

### Splice site strength improves when flanking splice sites are weakened

**Fig. 6:**
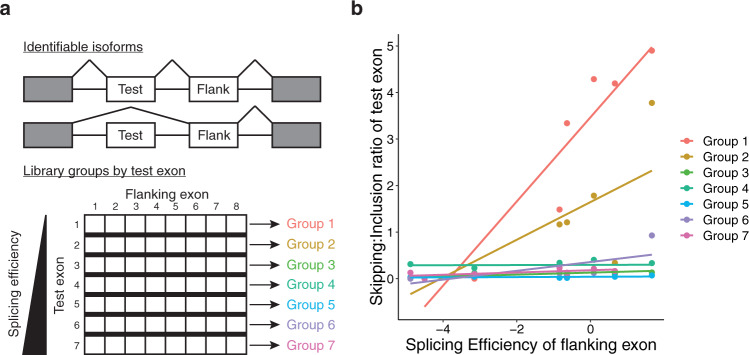
Higher-order effects of flanking exons on splicing. **a** Spliced isoforms that can be distinguished in RNA-seq. The minigene library was subdivided into seven groups, corresponding to the rows of the matrix. Each matrix element is a unique species, or pairing of test exons with flanking exons. Thus, each library group represents the pairings of one test exon with all flanking exons. The test exon used in Group 1 has the lowest splicing efficiency of all of the test exons, while the one in Group 7 has the highest splicing efficiency. **b** Skipping of the test exon is plotted as a function of the splicing efficiency of the flanking exon. Colors correspond to the library group of each species in panel (**a**).

Exon skipping is a competitive process in which a 5’ss often has a choice of two or more potential 3’ss, and vice versa. The splice site that is ultimately selected can be modulated either by enhancing the affinity for the desired site, or by repressing the competing site(s). The enhancement of splicing events by the suppression of competing splice sites can be found in natural alternative splicing regulation. For example, PTBP1 normally acts as a splicing repressor when it binds near either splice site of an exon; however, it can also act as an enhancer when it binds near the competing 3’ss of the flanking exon^25^. In the latter case, PTBP1 may indirectly enhance splicing in the first exon by suppressing one of its competing splice sites. We term this phenomenon enhancement by suppression of competitors (ESCo). ESCo supports the idea that neighboring sites of a particular class (e.g., 3’ss or 5’ss) are effectively in competition with each other. Using exonic splicing efficiency as a proxy for splice site strength, a minigene library was constructed to measure how splicing in a test exon can be affected by the splicing efficiency of a downstream flanking exon. Exons were selected from an independent single-exon construct previously used to determine their splicing efficiencies^3^. Each minigene species represents a unique pairing of splicing efficiencies. The library was divided into groups such that the first exon of the pair in each species of the group was identical (Fig. 6a). This assay, therefore, measures how these test exons splice differently when the splicing efficiency of their flanking exons change. For example, Group 1 contains the weakest test exon. When paired with weaker flanking exons, test exon inclusion is enhanced. ESCo is more readily detected with weak exons (Groups 1 and 2) and becomes insignificant when exons are sufficiently strong (Groups 3–7) (Fig. 6b). Although the GBM classifier suggests that exonic features such as splice site strength are informative when predicting splice-disrupting variants, flanking splice sites also influence inclusion in a predictable way.

### Gradient boosting classifier determines which exons are hotspots

**Fig. 7:**
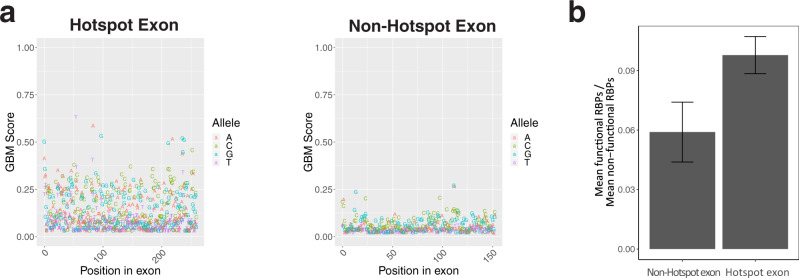
GBM classifier determined hotspot exons. **a** GBM predictions are plotted for all possible variants in an example hotspot exon (left) and non-hotspot exon (right). Variants with a GBM score > 0.5 are classified as splice-disrupting variants. The *x*-axis represents the nucleotide position across the example exons. **b** Reanalysis of RBP binding between hotspot (*n* = 1400 exons) and non-hotspot (*n* = 102,600 exons) exons determined by the GBM classifier. Data are presented as mean ± SD.

Since there are numerous patterns that describe hotspot exons, it is important to classify them using a unified, principled method. The GBM classifier for splice-disrupting variants is a fitting method that combines the critical features of hotspot exons, such as splice site usage and *cis*-element scores. Although the classifier is designed to predict splice-disrupting variants, it can also be extended to operationally define hotspot exons. For example, in hotspot exons, the GBM classifier generally reports higher scores for each possible exonic mutation (Fig. 7a). Therefore, an exon is defined as a hotspot exon if more than 10% of all possible exonic variants are predicted to disrupt splicing (GBM score > 0.5) (Supplementary Data 2). To ensure that this classification method remained faithful to previous results, the analysis of functional RBPs was revisited. Exons that were considered hotspots using the GBM classifier still showed a greater amount of functional RBPs relative to non-functional RBPs (Fig. 7b). These results suggest that the GBM classifier is a sufficient tool for determining hotspot exons.

If the results from the GBM classifier are meaningful, they should imply that hotspot exons relate to functional outcomes. In lieu of using functional assays, this relation could be inferred from the way that different exons tolerate variants. For example, if hotspot exons are important for function, they should not tolerate variants as well as other exons. Variants discovered in the ExAC project were used to compare tolerance among exons^26^. We found that those exons classified as hotspot exons have a significantly lower number of variants per base than those which are non-hotspot (one-sided *t* test, *p* <  0.05, *n* = 3000). An exon’s tolerance for variants could be impacted by the gene in which it is contained. Genes with higher HI scores are less tolerant of variants, and they largely resemble disease genes. When the list of hotspot exons was limited only to genes with high HI scores, these exons were still significantly less tolerant of variants (*n* = 1400). Hotspot exons therefore contribute to gene function, and they are located in disease genes.

## Discussion

As the cost of deep sequencing declines, the backlog of unclassified variants continues to grow. Since up to one-third of disease-causing variants disrupt splicing, it is crucial to determine the splice-disrupting potential of novel variants. Numerous computational approaches have been undertaken to predict exon skipping and other alternative splicing events. Some of these early methods focused only on describing splice site sequences through mathematical models^27,28^. Others focused on the impact of a mutation on enhancer or silencer motifs (ESE/ESS) by analyzing in vitro selection data^29–31^. Later, as splicing reporter assays developed, additional approaches yielded new scores for short sequences based on empiric data^22,32,33^. The GBM model developed here aggregates features from many of these approaches as well as features from whole genome annotation data, in order to predict the effect of exonic variants on exon skipping (Fig. 1). By performing feature selection on this model, groups of features were prioritized according to their contribution to predictive power. Although much attention has been paid to ranking *cis*-element motifs, the motif features used contributed little predictive power (Fig. 2). Instead, a major portion of the predictive power was influenced by exonic features. Empiric measures of exonic splicing strength, such as splice site usage and WT splicing outcomes in MaPSy, are especially important features. A recently developed neural network model that was trained on primary sequence alone also found that its predictions largely corresponded with chromatin binding states of exons^34^. The dominance of exon-level features in predicting splice disrupting variants led us to hypothesize the existence of hotspot exons. Hotspot exons, which contain larger numbers of splice-disrupting variants that could be linked to diseases, represent targetable loci for novel therapeutics. Variants discovered in hotspot exons through deep sequencing could be prioritized for follow-up studies to determine their pathogenicity. Such studies, like MaPSy, would benefit from this prioritization because they rely on multiplex PCR. As the number of PCR amplicons rises, it becomes more difficult to accurately measure each one. If the analysis is limited to exons whose splicing is predicted to be affected by perturbations, the precision in these downstream assays will improve.

An expedient method of identifying potential hotspot exons is to look at the variants reported in those exons that have associated splicing measurements. Because the GBM classifier points to exonic features and not mutation features as the major drivers of splicing, it is not surprising that variants in the same exon tended to have similar effects on splicing (Fig. 3). However, some hotspot exons may not yet have any reported splice-disrupting variants. Since splice-disrupting variants often interfere with *cis-*elements, RBP binding patterns in exons could be an alternative supplementary approach to identifying hotspot exons. Hotspot exons require larger numbers of functional RBP binding sites (Fig. 4), suggesting that most of its *cis*-elements are essential for exon inclusion. In addition, the more binding events an exon depends on, the less often they will all occur in the same transcript, resulting in more skipping. Therefore, a weakly splicing exon should signal that it is indeed a hotspot exon.

Splice site usage is an empiric measure of splicing strength that is derived from RNA-seq data (Fig. 5a). Splicing strength can also be measure by percent spliced-in (PSI), but this metric does not distinguish between splice sites. A recent study utilizing empirical data to interrogate the effect of mutations on splicing, found that mutations are more likely to disrupt splicing when their wild-type exon has an intermediate PSI level^13^. Consistent with this prediction, splicing in exons with lower splice site usage levels are more likely to be disrupted by mutations and other perturbations (Fig. 5b). Moreover, the same exons that are sensitive to mutations are also sensitive to drugs, which might reverse their effects on splicing. Splice site usage or PSI levels could therefore be used in conjunction with RBP knockdown data to identify potential hotspot exons. Because there are multiple criteria that determine hotspot exons, it is necessary to construct a unified method for calling them. The GBM classifier was the most natural approach, as it condenses many of these features into one score. When determining hotspot exons this way, previous results remained unaffected (Fig. 7).

Splice site usage depends on the surrounding sequence context in the pre-mRNA transcript, not just the features of the exon. A study using minigenes showed that similar 5’ss sometimes led to large differences in PSI depending on the minigene used^5^. The 5’ss most affected tended to have intermediate PSI levels (20–80%). The phenomenon we call Enhancement by *Suppression of Competitors* also suggests that splice site usage depends on the strength of a competing flanking splice site. In minigenes of competing exons with various combinations of splicing efficiencies, ESCo only occurred in test exons that were sufficiently weak (Fig. 6). ESCo could be a reliable mechanism for targeting aberrant splicing with novel therapeutics. Instead of directly targeting an exon with a splice-disrupting variant, drugs could target flanking exons with the intention of reducing their recognition. These drugs would not be limited to single alleles as they could indirectly target aberrant splicing for any number of variants by disrupting splicing in flanking exons. Analysis of the effect of amiloride on splicing demonstrates that a drug can disrupt splicing in numerous exons. Oligos targeting a hotpot’s two flanking exons could rescue mutations in hotspot exons. Many other drugs and small molecules have also been shown to disrupt splicing^9–12^. Drug discovery efforts are increasingly focused on targeting RNA. These compounds could be redeployed in precision medicine using the principle of ESCo. Splicing targets for these drugs could be discovered by screening with deep sequencing. If an FDA-approved drug is found to rescue a splicing disorder, it could be repurposed for a fraction of the development cost of a novel therapeutic. Conversely, when a drug is found to exacerbate a splicing disorder, a warning could be placed on that drug for relevant patients. Screening drugs for their effects on splicing will open avenues for precision medicine, in which rare diseases could be treated at a reasonable cost.

## Methods

### Prediction model

HGMD variants assayed in the MaPSy study were used to train the prediction model. Training features were calculated using in-house scripts **(**Table S1**)**. The ‘gbm’ R package with the following parameters was used to train the model: distribution: Bernoulli, number of trees: 1000, learning rate: 0.05, interaction depth: 3, bag fraction: 0.5, minimum number of observations in a node: 10, cross-validation folds: 3. The additional Random Forest model was trained using the “randomForest” R package with default parameters. 70% of the variants were used for training and the remaining 30% for evaluation. All in-house source code for this paper can be found at https://github.com/dtglidden/hotspot-exon-paper.

The trained GBM model outputs a value between 0 and 1 for each inputted sample, with the value representing the model’s confidence that a given mutation has an effect on the splicing of its host exon. A value close to 1 represents a mutation which affects splicing with high confidence, while a value close to 0 indicates high confidence that the mutation has no effect on splicing. The distribution of model predicted scores formed a bimodal “U-shaped” distribution clustered around 0 and 1, so for all further analyses, a value of 0.5 was chosen as the cutoff between splice-altering and no-effect mutations. Given the scarcity of predictions with intermediate values, any cutoff between 0.2 and 0.8 would have been functionally identical, but 0.5 was kept for simplicity.

The features required for the machine learning models can be broken into two categories: genomic features and mutation features. Genomic features are those that do not depend on the presence of any mutations, and may therefore be calculated and stored in advance. Mutation features are precomputed for the MaPSy alleles used to train the models. Newly submitted mutations for prediction have their features calculated at the time of submission. The outcome variable, allelic ratio, is calculated by the following formula (Eq. 1)

1 $${{\rm{log }}}_{2}\left(\frac{{m}_{o}/{m}_{i}}{{w}_{o}/{w}_{i}}\right)$$

where the subscripts $o$ and $i$ correspond to the output (spliced) and input (unspliced) fractions of mRNA respectively, and $m$ and $w$ correspond to the mutant and wild-type species, respectively.

### RBP binding and knockdown analysis

For 35 and 31 RBPs known to affect splicing, we obtained eCLIP binding peak data in the K562 and HepG2 cell lines, respectively, from the ENCODE project. The file identifiers along with the corresponding RBPs and cell lines for the peak files used are listed in the table below. Data from knockdown RNA-seq experiments for these RBPs were obtained from the Graveley lab and are also available on ENCODE (Table S2) (https://www.encodeproject.org/awards/U41HG009889/). In order to classify which binding peaks have evidence supporting an effect on splicing for a nearby exon, we performed an integrated analysis of the eCLIP and RBP knockdown data as follows. For the knockdown experiment of RBP_i_, we used rMATS to calculate differential splicing results for the comparison between the unperturbed and RBP_i_-depleted conditions^35^. Then, for every exon in the rMATS results, we searched for binding peaks of RBP_i_ within a window from the end of the upstream exon to the start of the downstream exon. If the exon was differentially spliced at an FDR < 0.1, then any such peaks are labeled as splice-affecting for that exon; otherwise, they are labeled as non-splice-affecting sites.

In order to assess how binding sites are distributed across exons, we constructed a model of the expected number of binding sites per exon through sampling of the subset of non-splice-affecting sites. 1000 samples without replacement (n = the number of splice-affecting sites for the cell line in question) were taken from this set of binding sites and estimates of the expected number of sites per exon were calculated from them. We then compared this expected distribution to the observed distribution of splice-affecting sites in order to obtain enrichment values for the number of exons with different amounts of splice-affecting RBP binding.

### Quantification of alternative splicing through switch score

The extent to which the exons in this analysis are alternatively spliced was assessed by calculating switch scores for each exon. The switch score is the maximum pairwise difference in percent-spliced-in for an exon when comparing among a set of conditions. In this case, we calculated inclusion from RNA-seq data for 19 cell lines obtained from ENCODE (Table S2) to serve as a proxy for assaying tissue-specific splicing events. We used rMATS on every possible pair of cell lines to acquire each pairwise difference in inclusion level needed to calculate the switch score.

### Calculating splice site usage from RNA-seq data

The splice site usage of a 5′ or 3′ splice site is meant to estimate the proportion of transcripts from a gene that undergo a splicing event that utilizes that particular site. To accomplish this we analyze exon-exon junction reads obtained from mapping of RNA-seq data. For a given splice site, there are three categories of junction reads which go into calculating its splice site usage: (A) a read that has the splice site as one of the sides of the junction, (B) a read that spans the splice site (i.e., the junction is between a site that is upstream and one that is downstream of the site in question), and (C) for a 5′ (3′) splice site, a read with a junction that has its 5′ (3′) end in the downstream (upstream) intron. From the counts of these three classes of reads, splice site usage is defined as $\frac{A}{A+B+C}$. In order to mitigate the corruption of this metric by the false positive splice junctions frequently output by RNA-seq aligners, we only considered junction reads that contained splice sites present in the GENCODEv29 annotation. If there were multiple RNA-seq replicates for a particular cell line or condition, we collapsed the junction read counts from all replicates before calculating splice site usages.

### Identification of hotspot exons with GBM model

In order to identify all hotspot exons in the genome, we first generated predictions from the trained GBM model for each possible exonic mutation. We then designated those exons where greater than 10% of all possible mutations were predicted to disrupt splicing as hotspot exons, and all others as non-hotspot exons. Biologically, we define hotspot exons as those which are sensitive to perturbations even outside of the canonical splice sites. In the context of the prediction model, this definition implies that an abnormally high proportion of all possible mutations should affect splicing, which motivated our use of a proportion-based threshold, rather than average GBM score. The value of 10% was chosen as it maximized the difference in the number of variants per base in the groups above and below the cutoff while retaining as many exons as possible. Therefore, we found it to strike a good balance between precision and recall of hotspot exon identification.

Variants per base were calculated for each exon from mutation data collected by ExAC according to the following formula (Eq. 2).

2 $$\frac{{\rm{number}}\,{\rm{of}}\,{\rm{distinct}}\,{\rm{observed}}\,{\rm{variants}}\,{\rm{in}}\,{\rm{an}}\,{\rm{exon}}}{{\rm{length}}\,{\rm{of}}\,{\rm{exon}}\left({\rm{bp}}\right)}$$

The data was downloaded from https://gnomad.broadinstitute.org/downloads. The number of variants per base is indicative of how well an exon tolerates mutations, with low values indicating exons that are very sensitive to perturbations. A true hotspot exon should have a low number of variants per base, as a large proportion of mutations would be deleterious and selected against. To validate our approach to identifying hotspot exons and to select the best cutoff, we compared the variants per base across those exons with a proportion of splice-altering mutations above the cutoff (hotspot) to those below the cutoff (non-hotspot) across a range of different cutoffs. In order to accurately compare the variants per base across the two groups, the non-hotspot exons were down-sampled to the number of hotspot exons. To minimize bias, the compared non-hotspot exons were selected from the same set of genes that the hotspot exons were located in, and size-matched as closely as possible. This analysis was performed both with and without filtering exons by haploinsufficiency score, which was performed as follows: Exons were assigned the HI score of the transcript they were most often transcribed in. The median HI score of all genes was calculated (0.53). Exons with an HI score below the median were then filtered out. Haploinsufficiency scores were downloaded from https://github.com/HAShihab/HIPred.

### Cell culture

HEK293T cells (ATCC) were cultured with Dulbecco’s Modified Eagle Medium (DMEM) + 10% fetal bovine serum (Invitrogen) and 1% penicillin/streptomycin (Sigma), and incubated at 37 °C, 5% CO_2_. Cells were passaged at 70–90% confluence via trypsinization, and passage number was kept low. Cells were checked periodically for mycoplasma contamination.

### Drug treatment analysis

HEK293T cells were grown to >70% confluence. One day prior to drug treatment, cell culture media was replaced with a fresh batch that did not contain antibiotics in order to minimize the effect of the antibiotics on splicing. Afterwards, 300 µg/mL of amiloride (Sigma) was dissolved into the media. 6 h later, total RNA was extracted using TRIzol (Invitrogen). RNA was converted to cDNA using SuperScript IV (New England Biolabs) and analyzed by deep sequencing (Illumina HiSeq 2500, 126-bp paired end reads, polyA+ fraction).

### Minigene assay

Selected exons previously assayed with MaPSy (Table S3) were amplified via PCR (Table S4) out of 3-exon minigene plasmids and ligated via Gibson assembly (NEB) to create 4-exon linear minigene constructs. The upstream flanking sequence included a CMV enhancer, promoter, and 71 bp of the Ad81 exon, and the downstream flanking sequence included ACTN1 exon 16 and a bGH polyA tail (see “MaPSy in vivo assays” section of the “Methods” section in Soemedi et al. (2017), for a more complete description of the library species). Two PCR amplifications were performed on the plasmids, creating two 2-exon species to be ligated. First, the CMV enhancer, promoter, Ad81 exon, and the test exon with additional downstream intronic sequence were amplified as one species (library part 1). The reverse primer included a 20 bp extension sequence for future ligation with Gibson assembly. Second, the same test exon with addition upstream sequence, ACTN1 exon 18, and the polyA tail were amplified as another species (library part 2). The forward primer included the same 20 bp extension sequence such that Gibson assembly would produce a 4-exon construct with two of the test exons in the middle, spaced apart by sufficient intronic sequence. Ligation reactions were performed such that each sample contained one species from library part 1 ligated to all possible species in library part 2 (8 species per sample). Minigene species were transfected with Lipofectamine 3000 (Invitrogen) for 24 h. Total RNA was extracted, and spliced isoforms were measured by RT-PCR. Samples were subject to deep sequencing (Illumina MiSeq, 150 bp paired-end reads).

### Reporting summary

Further information on research design is available in the Nature Research Reporting Summary linked to this article.

## Supplementary information

Supplementary Information

Supplementary Data 1

Supplementary Data 2

Reporting Summary

Description of Additional Supplementary Files

## Data Availability

We provide three publicly available resources for further study in splicing prediction and splice site usage. First, using our GBM model, we present splicing predictions for all possible exonic mutations in exons with measured splice site usage (Supplementary Data 1). Second, we provide average splice site usage data across 19 ENCODE cell lines for splice sites with low variance in usage. These data can be viewed as a public track on the UCSC Genome Browser (http://genome.ucsc.edu/s/dschmelt/KrasSpliceHub). Finally, we provide a list of all exons that we classify as hotspot exons in the HEK293T cell line (Supplementary Data 2). RNA-seq data from amiloride treatment of HEK293T cells are available in the NCBI gene expression omnibus (GEO) under accession GSE140786. RBP binding and knockdown data are available from the ENCODE project (https://www.encodeproject.org/awards/U41HG009889/). The data supporting the findings of this study are available from the corresponding authors upon reasonable request.

## Code availability

Code use for data analysis can be found at (https://github.com/dtglidden/hotspot-exon-paper).
