# Supplementary material for: Hotspot exons are common targets of splicing perturbations
Source: Nat Commun. 2021 May 12;12:2756. doi: 10.1038/s41467-021-22780-2 (PMC8115636; doi:10.1038/s41467-021-22780-2)
Supplement: Supplementary file 1 — Supplementary Information [file 41467_2021_22780_MOESM1_ESM.pdf]

Supplemental Material

Table S1: Features used for machine learning models.

feature	level	description
mutation_base_change	Mutation	The actual SNV (e.g G->T)
isWtPaired	Mutation	RNA−RNA hybridization state at the position of the mutation for the WT base
isMutPaired	Mutation	RNA−RNA hybridization state at the position of the mutation for the Mut base
mwdif_3score	Motif	Mut 3'SS Maxent score - WT 3'SS Maxent score
mwdif_5score	Motif	Mut 5'SS Maxent score - WT 5'SS Maxent score
avgDeltaEIScores	Motif	Average change in hexamer EI for all hexamers affected by the mutation
avgDeltaA3SSScores	Motif	Average change in Shendure A3SS score for all hexamers affected by the mutation
avgDeltaA5SSScores	Motif	Average change in Shendure A5SS score for all hexamers affected by the mutation
hek_ws	Exon	MaPSy in vivo spliced counts
hek_wu	Exon	MaPSy in vivo unspliced counts (input)
w3score	Exon	WT 3'SS Maxent score
w5score	Exon	WT 5'SS Maxent score
ss3usage	Exon	3'SS usage
ss5usage	Exon	5'SS usage
ei	Exon	Average EI score for all hexamers in the exon
nExons	Transcript	Number of exons in the canonical transcript
geneLength	Transcript	Full length of the gene

Table S2: ENCODE cell lines

A549
AG04450
BJ
CD14
CD20
GM12878
H1-hESC
HeLa-S3
HepG2
HMEC
HSMM
HUVEC
IMR90
K562
MCF-7
NHEK
NHLF
SK-N-SH
SK-N-SH-RA

Table S3: Test exons

Gene	Exon Number
**MLH1**	15
**ACADS**	9
**ASL**	8
**CPS1**	24
**COL1A1**	37
**BRCA1**	22
**BTK**	10
**OPA1**	12

Table S4: Oligonucleotides

Name	Sequence
**Library species part 1 forward**	5’-CCT ATG GTG CAC TCT CAG TAC AAT CTG C-3’
**Library species part 1 reverse**	5’-TCA CAT CAC CAT GCC TGG CTA ACA ACA AGG CGA CTT TCA G-3’
**Library species part 2 forward**	5’-AGC CAG GCA TGG TGA TGT GAC TCT CAA AAG CGG GCA TGA C-3’
**Library species part 2 reverse**	5’-GCT GGT TCT TTC CGC CTC AG-3’

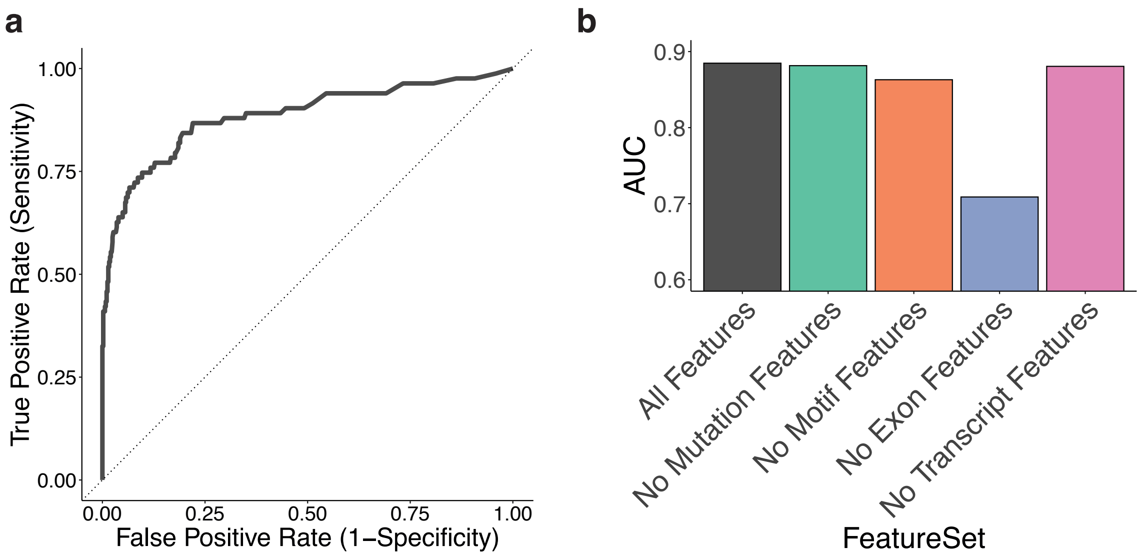

Figure S1: Prediction performance for Random Forest model. (a) Receiver-operator characteristic (ROC) curve for the Random Forest model trained on all available features. **(b)** Area under the curve (AUC) for several iterations of the model using subsets of available features.

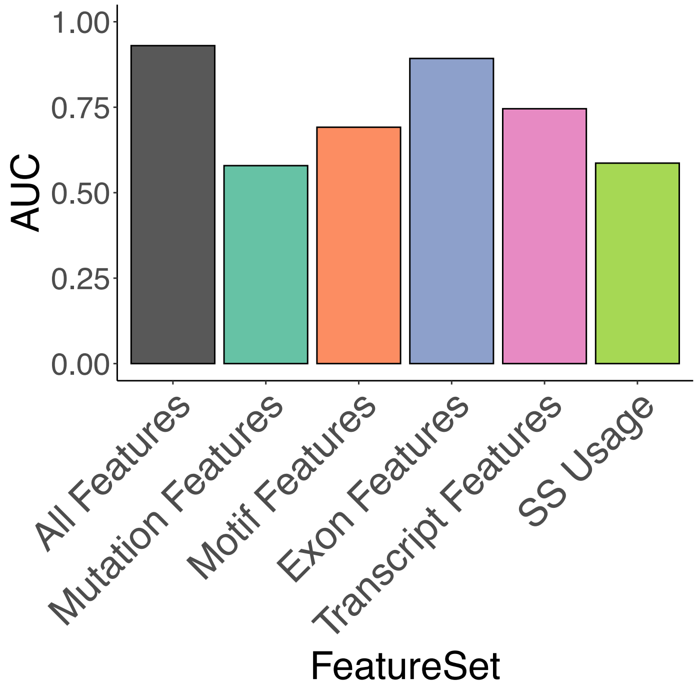

Figure S2: Prediction performance of individual feature groups for GBM model. Bar plot of the area under the curve (AUC) for different iterations of the GBM classifier trained on one feature group. In addition, the AUC for the GBM models trained only on SS usage (an exon feature) is shown.

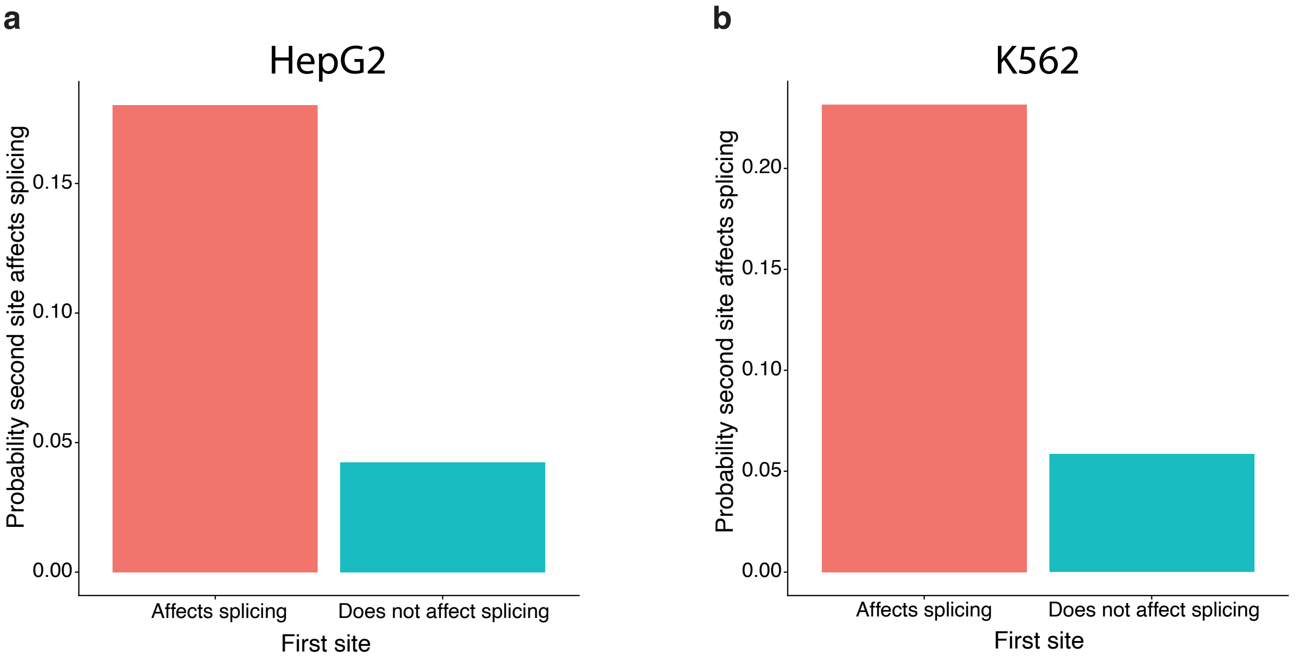

Figure S3: Presence of a functional RBP site predicts subsequent RBP sites.

Two RBP sites were randomly sampled for exons with more than one site and the conditional probability that the second site is functional given the functionality of the first was estimated.

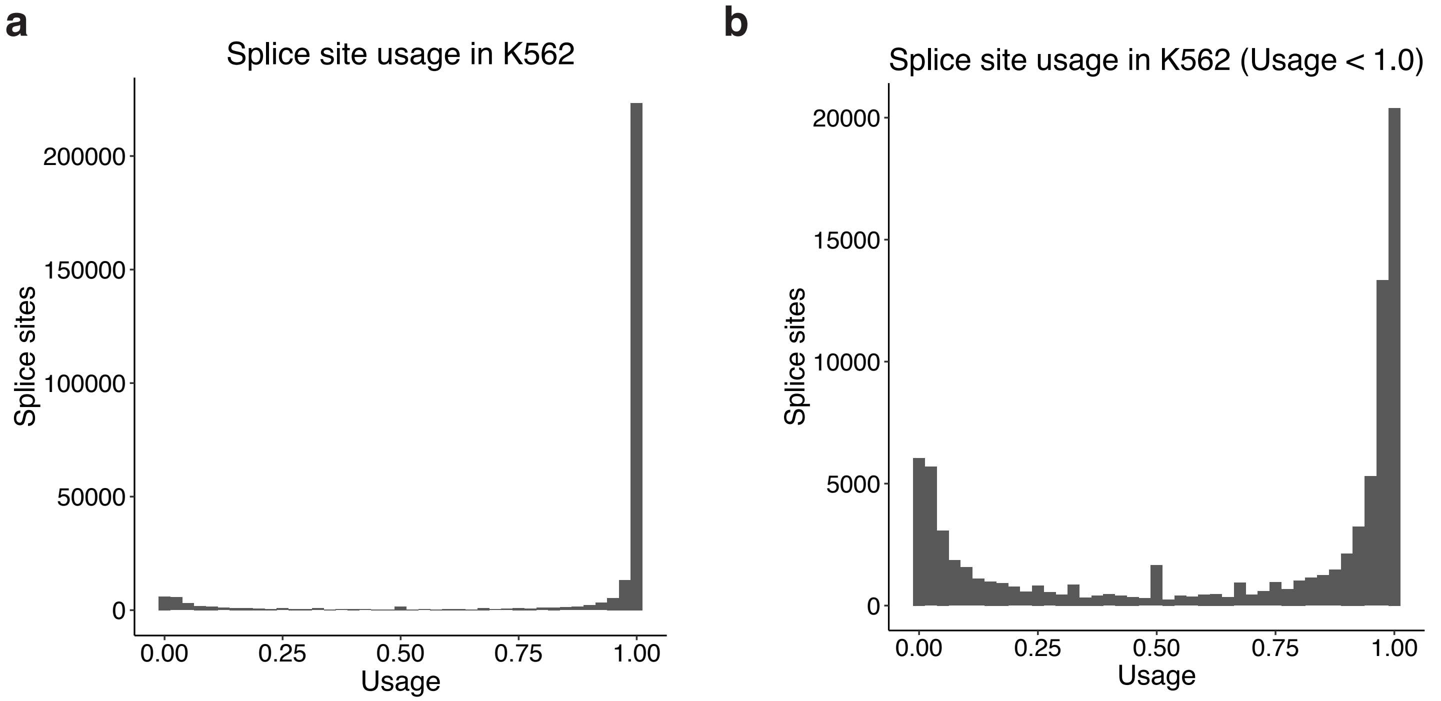

Figure S4: Distribution of splice site usage in K562 cells. (a) Splice site usage for all splice sites in the K562 cell line is plotted. **(b)** Because of the abundance of splice sites with a usage of 1, a second histogram is plotted, which excludes these splice sites.

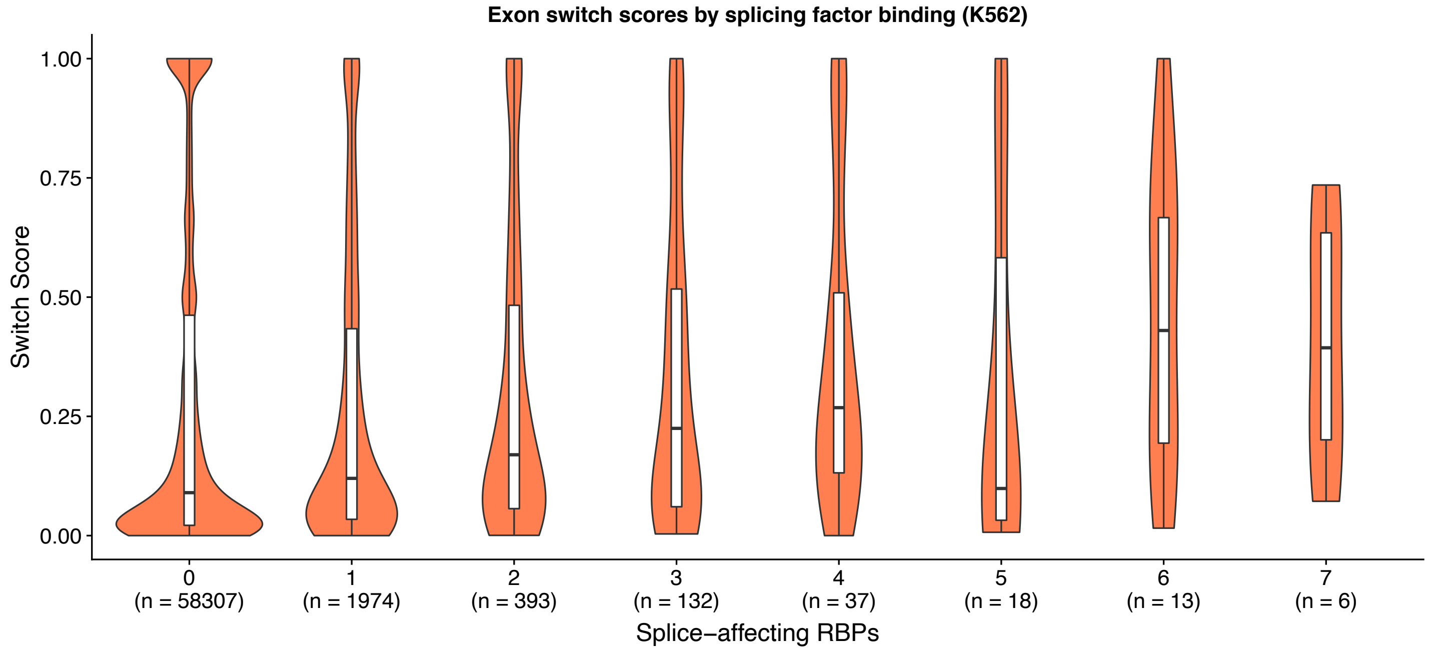

Figure S5: Relationship between RBP-binding and alternative splicing as measured by exon switch score. Quantification of exon binding events and switch score described in methods. Each boxplot represents the quartile ranges of switch scores, such that the lower range of each box is the 25th percentile, the upper range is the 75th percentile, and the line in each box is the center at the median. Whiskers extend from the 1st to the 99th percentile.

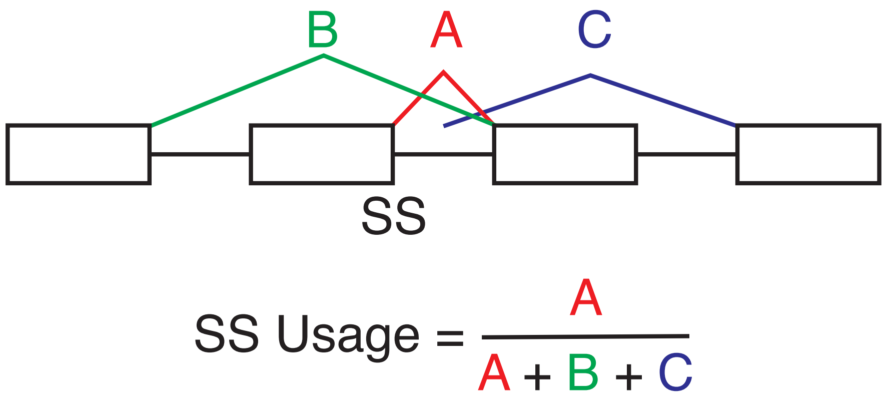

Figure S6: Calculation of splice site usage. RNA-seq reads are categorized based on whether they use a splice site (A, red) skip a splice site (B, green) or use a cryptic splice site in the intron adjacent to the splice site (C, blue). The number of reads in each category is used to calculate splice site usage with the formula depicted.

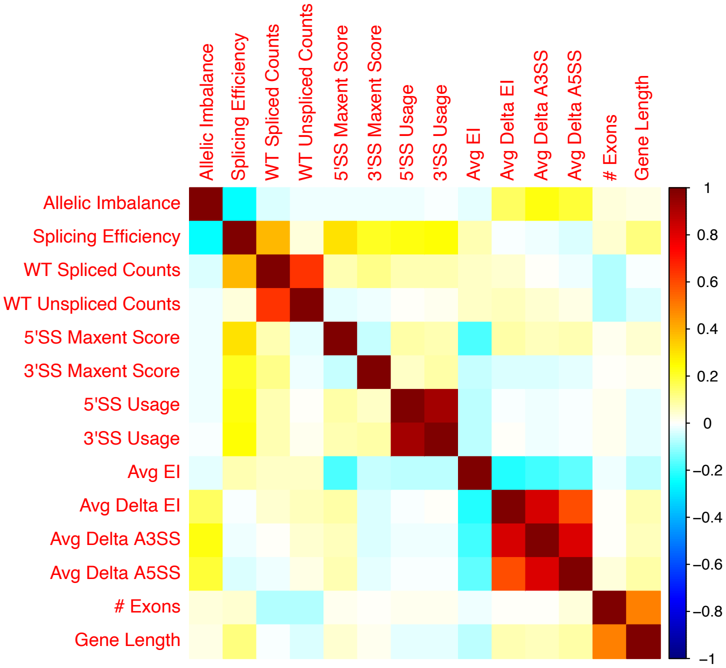

Figure S7: Correlation of Prediction Features. A heatmap of the correlation matrix shows the Pearson's R values for each pair of features (two-sided test). M/W Ratio is the outcome variable used in the prediction model.
