# Supplementary material for: Hotspot exons are common targets of splicing perturbations
Source: Nat Commun. 2021 May 12;12:2756. doi: 10.1038/s41467-021-22780-2 (PMC8115636; doi:10.1038/s41467-021-22780-2)
Supplement: Supplementary file 4 — Reporting Summary [file 41467_2021_22780_MOESM4_ESM.pdf]

nature research

Corresponding author(s): William Fairbrother

Last updated by author(s): Feb 3, 2021

Reporting Summary

Nature Research wishes to improve the reproducibility of the work that we publish. This form provides structure for consistency and transparency in reporting. For further information on Nature Research policies, see Authors & Referees and the Editorial Policy Checklist.

Statistics

For all statistical analyses, confirm that the following items are present in the figure legend, table legend, main text, or Methods section.

n/a	Confirmed	
☐	☒	The exact sample size (*n*) for each experimental group/condition, given as a discrete number and unit of measurement
☐	☒	A statement on whether measurements were taken from distinct samples or whether the same sample was measured repeatedly
☐	☒	The statistical test(s) used AND whether they are one- or two-sided<br>*Only common tests should be described solely by name; describe more complex techniques in the Methods section.*
☒	☐	A description of all covariates tested
☒	☐	A description of any assumptions or corrections, such as tests of normality and adjustment for multiple comparisons
☐	☒	A full description of the statistical parameters including central tendency (e.g. means) or other basic estimates (e.g. regression coefficient) AND variation (e.g. standard deviation) or associated estimates of uncertainty (e.g. confidence intervals)
☐	☒	For null hypothesis testing, the test statistic (e.g. *F*, *t*, *r*) with confidence intervals, effect sizes, degrees of freedom and *P* value noted<br>*Give P values as exact values whenever suitable.*
☒	☐	For Bayesian analysis, information on the choice of priors and Markov chain Monte Carlo settings
☒	☐	For hierarchical and complex designs, identification of the appropriate level for tests and full reporting of outcomes
☐	☒	Estimates of effect sizes (e.g. Cohen's *d*, Pearson's *r*), indicating how they were calculated

Our web collection on statistics for biologists contains articles on many of the points above.

Software and code

Policy information about availability of computer code

Data collection	No software was used
Data analysis	Custom scripts for data analysis were written in either the R or Python programming language. These can be found at https://github.com/dtglidden/hotspot-exon-paper. The R packages 'gbm' and 'randomForest' which are cited in the main text, are versions 2.1.5 and 4.6-14 respectively.

For manuscripts utilizing custom algorithms or software that are central to the research but not yet described in published literature, software must be made available to editors/reviewers. We strongly encourage code deposition in a community repository (e.g. GitHub). See the Nature Research guidelines for submitting code & software for further information.

Data

Policy information about availability of data

All manuscripts must include a data availability statement. This statement should provide the following information, where applicable:

- Accession codes, unique identifiers, or web links for publicly available datasets
- A list of figures that have associated raw data
- A description of any restrictions on data availability

We provide three publicly-available resources for further study in splicing prediction and splice site usage. First, using our GBM model, we present splicing predictions for all possible exonic mutations in exons with measured splice site usage. Second, we provide average splice site usage data across 19 ENCODE cell lines for splice sites with low variance in usage. These data can be viewed as a public track on the UCSC Genome Browser (http://genome.ucsc.edu/s/dschmelt/KrasSpliceHub). Finally, we provide a list of all exons that we classify as hotspot exons in the HEK293T cell line. Additional sequencing data are available in the NCBI gene expression omnibus (GEO) under accession GSE140786.

Field-specific reporting

Please select the one below that is the best fit for your research. If you are not sure, read the appropriate sections before making your selection.

☒ Life sciences ☐ Behavioural & social sciences ☐ Ecological, evolutionary & environmental sciences

For a reference copy of the document with all sections, see nature.com/documents/nr-reporting-summary-flat.pdf

Life sciences study design

All studies must disclose on these points even when the disclosure is negative.

Sample size	Sample sizes were determined based on publicly-available datasets in the form of RNA-seq data. Studies were not amenable to power analyses to determine sample sizes in advance. After processing the RNA-seq data for splicing events, statistical significance for differential splicing events was based on the number of sequencing reads covering exons and exon-exon junctions. Differential splicing events that were not statistically significant were not used.
Data exclusions	No data were excluded.
Replication	Relevant differential splicing events found in RNA-seq data were validated with RT-PCR, which was then used in subsequent samples to confirm reproducibility.
Randomization	When designing our experiments, control and treatment samples came from the same original stock of cells, and were subject to the same conditions. No further randomization was required.
Blinding	Blinding was not relevant to our study. Our experiments involved treating tissue culture cells with a drug or nucleic acid transfection. No blinding is possible in this design.

Reporting for specific materials, systems and methods

We require information from authors about some types of materials, experimental systems and methods used in many studies. Here, indicate whether each material, system or method listed is relevant to your study. If you are not sure if a list item applies to your research, read the appropriate section before selecting a response.

Materials & experimental systems

n/a	Involved in the study	
☒	☐	Antibodies
☐	☒	Eukaryotic cell lines
☒	☐	Palaeontology
☒	☐	Animals and other organisms
☒	☐	Human research participants
☒	☐	Clinical data

Methods

n/a	Involved in the study	
☒	☐	ChIP-seq
☒	☐	Flow cytometry
☒	☐	MRI-based neuroimaging

Eukaryotic cell lines

Policy information about cell lines

Cell line source(s)	ATCC (HEK 293T)
Authentication	Authenticated by morphology
Mycoplasma contamination	Not tested
Commonly misidentified lines (See ICLAC register)	No commonly misidentified cell lines were used
