# Supplementary material for: Hotspot exons are common targets of splicing perturbations
Source: Nat Commun. 2021 May 12;12:2756. doi: 10.1038/s41467-021-22780-2 (PMC8115636; doi:10.1038/s41467-021-22780-2)
Supplement: Supplementary file 5 — Description of Additional Supplementary Files [file 41467_2021_22780_MOESM5_ESM.pdf]

Description of additional supplementary files

Title: Supplementary Data 1

Description: Splicing predictions for all possible exonic mutations in the human genome (hg19 assembly). Scores range from 0-1, where 0 is the least likely to affect splicing, and 1 is the most likely to affect splicing.

Title: Supplementary Data 2

Description: All exons in the human genome (hg19 assembly) that are classified as hotspot exons. Classifications are based on splicing levels in the HEK293T cell line.
